# Electric Control of the Hall effect in Pt/Bi_0.9_La_0.1_FeO_3_ bilayers

**DOI:** 10.1038/srep20330

**Published:** 2016-02-03

**Authors:** Rongli Gao, Chunlin Fu, Wei Cai, Gang Chen, Xiaoling Deng, Hongrui Zhang, Jirong Sun, Baogen Shen

**Affiliations:** 1 School of Metallurgy and Materials Engineering, Chongqing University of Science and Technology, Chongqing, 401331, China; 2 Beijing National Laboratory for Condensed Matter Physics and Institute of Physics, Chinese Academy of Science, Beijing 100190, China

## Abstract

Platinum metal, being nonmagnetic and with a strong spin-orbit coupling interaction, has been deposited on weak ferromagnetic Bi_0.9_La_0.1_FeO_3_ thin films. The Hall effect is studied as a function of the polarization direction of multiferroic Bi_0.9_La_0.1_FeO_3_ thin films, as well as magnetic field (***H***) and temperature (***T***). For the two polarization directions, besides the obvious difference of the anomalous Hall resistance R_AH_, it increases sharply with decreasing temperature, and even changes sign, thus violating the conventional expression. This observations indicate local magnetic moments in Pt caused by the local electric fields at the interface of Bi_0.9_La_0.1_FeO_3_ films. Also, possible proximity effects and induced magnetic ordering in Pt on weak ferromagnetic Bi_0.9_La_0.1_FeO_3_ thin films of both upward and downward polarization states may exist and their contribution to the spin-related measurements should not be neglected.

It has been known that the Hall resistivity * ρH* in a ferromagnet^1^ has some extra contribution originated from the spontaneous magnetization, which is assumed and often observed experimentally to be fitted by


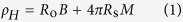


where *R*_*o*_ is the usual Hall coefficient, while *R*_*s*_ is called the anomalous Hall coefficient. The anomalous Hall effect (AHE = 4π*R*_*S*_*M*) has been useful in the investigation and characterization of itinerant electron ferromagnets^1^. AHE in ferromagnetic conductors has received renewed interest in recent years due to its close connection with spin transport phenomena^2^ and the controversial mechanisms^3^. It is assumed from formula (1) that the anomalous contribution is proportional to the magnetization ***M***and it is often observed experimentally. However, recent experiments have found a nonmonotonic dependence on ***M*** even including the sign change, which gives an important clue on the origin of the AHE. Besides, the quantitative analysis of the anomalous Hall effect has seldom been completed since its mechanism has not yet been established, and theories give much smaller values compared with the experiments.

Recently, it was reported that in addition to magnetic materials, the anomalous Hall effect was also observed in nonmagnetic Platinum(Pt) films contacted with ferromagnetic materials^4^,^5^. It has also been reported that the magnetic proximity effect can induce spin polarized electrons in Pt thin films^4^. Ferromagnetic behavior has also been extensively reported in nanoparticles and clusters of transition metals such as Pd and Pt, presenting exotic physical properties quite different from bulk characteristics. Since Pt has strong spin-orbit interaction, it has often been used as a spin current detector, many experiments on spin-current injection and detection have been performed using Pt/ferromagnetic(FM) systems^6^. It should be pointed that when a thin Pt film has been deposited on a thick FM metal, its conducting and magnetic properties are overwhelmed by those of the FM metal. However, the conducting properties of thin Pt films deposited on an insulator can be readily determined through transport measurements. If the insulator is also FM, one may even access the magnetic properties. Fortunately, BiFeO_3_(BFO) thin films is exactly an insulator and it shows simultaneous ferroelectric and magnetic ordering (weak ferromagnetic) at room temperature^7^,^8^,^9^,^10^, has been extensively studied due to the coupling between their dual order parameters. In other words, the magnetization can be controlled by polarization switching after applying external electric field and vice versa. Thus, it is of critical importance and interest to ascertain the anomalous Hall effect of Pt contacted with BFO films and whether this effect can be tuned by external electric field according to manipulating the magnetization via polarization reversal.

Herein, we report electric control of the Hall effect in Pt/Bi_0.9_La_0.1_FeO_3_ bilayers. We have observed polarization dependence of Hall effect and nonmonotonic dependence of AH resistivity on the temperature and magnetization, including a sign change around room temperature. Rather than the more complex description by intrinsic Hall effect, however, all the features observed can be interpreted within a simple model of bilayers consist of nonmagnetic and magnetic Pt films by considering the magnetic proximity effects (MPE) with FM characteristics or the local magnetic moments in Pt with the help of its large spin-orbit interaction caused by local electric fields at the interface of Pt. These results raise questions about the suitability of using BFO in controlling Hall effect.

## Experimental Process

Patterned Pt thin films with thickness ~2.5 nm have been deposited by pulsed laser deposition (PLD) on epitaxial Bi_0.9_La_0.1_FeO_3_(BLFO) films. The BLFO thin films with the thickness of ~400 nm were grown epitaxially on (001)-oriented SrTiO_3_ (STO) substrates with 10 nm La_0.7_Sr_0.3_MnO_3_(LSMO) as buffer layers at 650 ºC. The other deposition parameters are depicted in detail elsewhere^11^,^12^,^13^. The nominal thickness of the individual layers was calculated from sputtering time and rates. Structural characterization of the BLFO films was performed using X-ray diffraction (XRD), using M/s Bruker make D8-Discover x-ray diffractometer using Cu K_α1_ radiation. To perform Hall and resistance measurements simultaneously and to guarantee that the data for electrical transport (Hall and longitudinal resistivity) measurements were obtained from the same sample, masks were used to make patterned samples. Transport measurements were carried out on a Quantum Design PPMS-14H at 300–2 K. The magnetization of all samples measured by MPMS-XL, and the residual longitudinal offset in measured Hall voltage was excluded using its different symmetry against the magnetic field, ***H***, and the anomalous part (*ρ*_*xy*_) of the Hall resistivity was extracted according to the deviation of H from the normal (Lorentz force) H-linear component. The samples for x-ray diffraction, surface morphology and transport measurements have the dimension of 10 × 10 × 0.5 mm^3^.

## Results and Discussion

**Figure 1:**
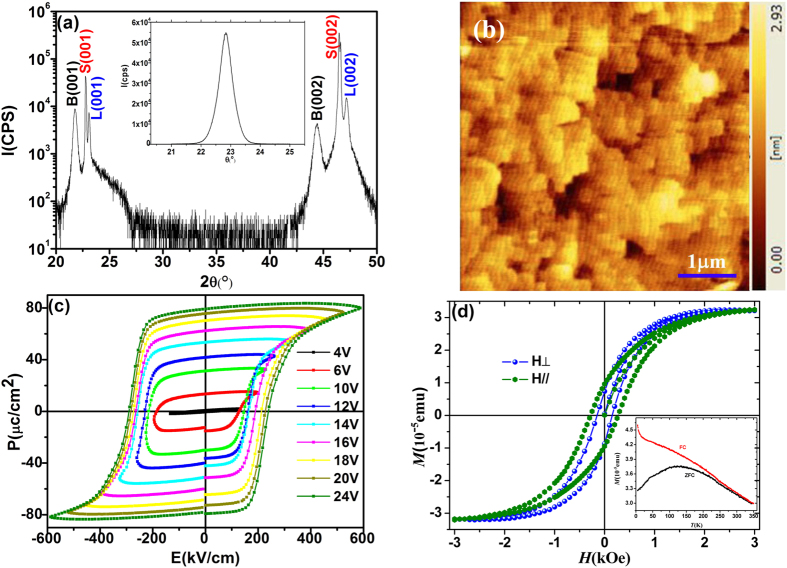
(**a**) XRD spectrum of BLFO/LSMO/STO films with 2θ in the range of 10~80 degrees, inset shows the rocking curve of (002) BLFO peak. (**b**) The Atomic force microscopy surface topography of a representative BLFO film, the scanning area is 5 × 5 μm^2^. (**c**) Polarizarion versus voltage (P-V) curves of BLFO films at room temperature. (**d**) Magnetization vs magnetic field curve for BLFO thin films of thickness 400 nm measured at 300 K along the in-plane and out of plane directions. The inset shows the temperature-dependent magnetization of BLFO films measured under a magnetic field of 500 Oe with both the ZFC and the FC conditions.

Figure 1(a) shows the θ-2θ XRD patterns of the BLFO thin films grown on LSMO-coated STO (001) substrates. Only the (00*l*) (*l* = 1, 2, 3) peaks of BLFO, LSMO and STO are visible, indicating a pure single-oriented perovskite BLFO phase. The out-of-plane *c* parameter for these films is *c* = 3.99 Å, very similar to the values obtained for undoped BFO films^13^,^14^. The BLFO layers have a top surface root mean square roughness of less than 0.35 nm over a scanning area of 5 × 5 μm^2^, as characterized by atomic force microscopy [Fig. 1(b)]. Figure 1(c) shows P-E hysteresis loops measured at a frequency of 10 kHz, it can be seen that the maximal remanent polarization (*Pr*) reaches nearly 80 μC/cm^2^ at the applied voltage of 24 V, which is slight bigger than the typical values of 60μC/cm^2^ obtained for BFO films^15^,^16^. Magnetization measurement for the BLFO thin film was conducted at RT and is plotted in Fig. 1(d), where a saturated magnetic loop (*Ms* = 3.0 × 10^−5 ^emu) is obtained with the magnetic field parallel and perpendicular the surface the slight difference between the two measure direction indicating the magnetic anisotropy of BLFO films.This *Ms* value is consistent with that of the single layer BLFO thin film that was directly deposited on STO substrate, indicating that the spin structure of the BLFO layer was not largely affected by the bottom LSMO layer in the bilayered BLFO/LSMO thin film. Inset of Fig. 1(d) shows the temperature-dependent magnetization of BLFO films measured under a magnetic field of 500 Oe with both the ZFC and the FC conditions. It reveals a typical spin-glass like behavior with a bifurcation of ZFC and FC curves developing progressively in the whole temperature range, indicating a strong thermal irreversibility. The spin-glass state generally occurs when positions of magnetic moments or signs of neighboring coupling appear in a random manner. This combination of magnetic randomness and mixed interactions cause frustration and stochastic disorder in the corresponding energy landscape.

**Figure 2:**
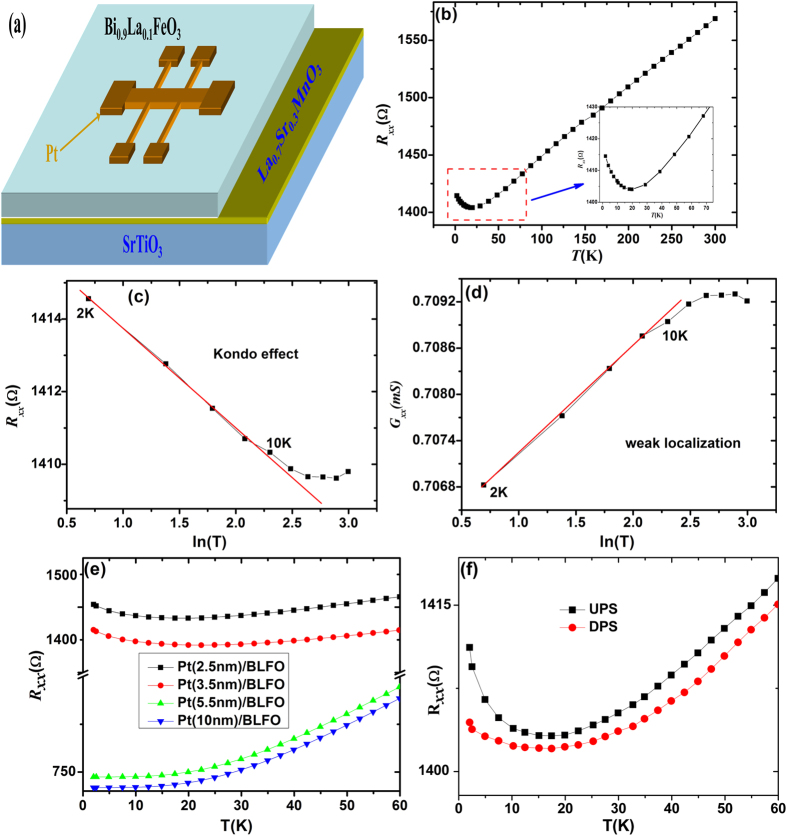
(**a**) The Schematic diagram of the patterned Hall bar on a substrate in the *xy* plane with ***H*** being perpendicular to this plane. (**b**) The temperature dependence of sheet resistance *R*_*xx*_ grown on BLFO thin film. (**c,d**) Are the resistivity vs ln*T* and and conductivity vs lnT for kondo effect and weak localization. (**e**) Is the temperature dependence of sheet resistance *R*_*xx*_ grown on BLFO with different thickness of Pt films. (**f**) Is the temperature dependence of sheet resistance *Rxx* grown on BLFO with upward polarization state and downward polarization state.

Figure 2(a) is the Schematic diagram of the patterned Hall bar on BLFO films in the *xy* plane with ***H*** being perpendicular to this plane. We show, in Fig. 2(b), the temperature (T) dependence of sheet resistance, the the resistivity of the Pt thin film decreases gradually with temperature reduction, which shows metallic T dependence. *R*_*xx*_ shows a minimum around 20 K and then increases with decreasing T below 20 K. The *R*_*xx*_ indicated insulating characteristics (*dR*_*xx*_*/dT* < 0) in the low-temperature region (below ~20 K). This resistance rise is almost proportional to lnT, indicating manifestation of the kondo effect or weak localization of electrons caused by dimensional effect. However, Quantitative analysis of the insulating resistance results shows that both conductance G_xx_ and resistance *R*_*xx*_ changes linearly with ln*T* at low temperatures, which can be seen in Fig. 2(c,d). In order to clarify which is the prime reason, the *R*_*xx*_ was measured under different thickness of Pt, as shown in Fig. 2(e), indicating thickness dependence, thus this phenomenon can be ascribed to the two-dimensional (2D) weak disorder-induced electron localization (EL)^17^. It was assumed that these ferromagnetic like characteristics strongly indicates strong proximity effects and induced magnetic ordering in Pt on magnetic insulators^5^. The induced Pt moment can be a result of the magnetic proximity effect (MPE). Figure 2(f) shows the temperature dependence of *R*_*xx*_ with upward polarization state (UPS, Negative voltage of −24 V was applied to Pt top electrode) and downward polarization state (DPS, Negative voltage of +24 V was applied to Pt top electrode) of BLFO films. We find that the MPE is obviously affected by polarization orientation, which indicates that the the magnetism of Pt deposited on magnetic BLFO films is able to be controlled by electric field.

**Figure 3:**
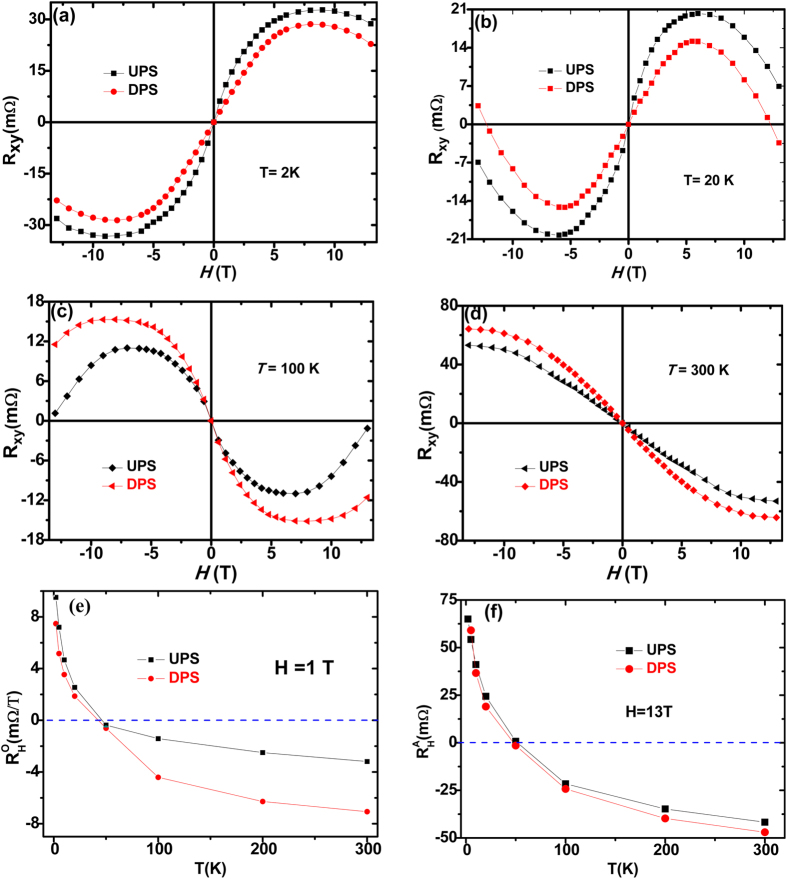
Representative field-dependent Hall resistivity curves measured with upward polarization state and downward polarization state. (**a**) Field-dependent Hall resistivity curves measured with upward polarization state and downward polarization state at 2 K. (**b**) Field-dependent Hall resistivity curves measured with upward polarization state and downward polarization state at 20 K (**c**) Field-dependent Hall resistivity curves measured with upward polarization state and downward polarization state at 100 K. (**d**) Field-dependent Hall resistivity curves measured with upward polarization state and downward polarization state at 300K. (**e**) Is the temperature-dependent ordinary Hall coefficient at the magnetic field is 1 T for BLFO film with upward polarization state and downward polarization state. (**f**) Is the temperature dependence of anomalous Hall resistivity *R_AH_* at H = 13 T for BLFO film with upward polarization state and downward polarization state. The solid lines are guides to the eye. With decreasing temperature, R_AH_ changes sign from negative to positive values.

The Hall resistivity, *ρ*_*xy*_, for UPS and DPS measured in a magnetic field –13T to 13T magnetic field at different temperatures were shown in Fig. 3(a–d), obvious difference can be seen between these two polarization directions throughout the whole temperature range. For this two directions, the resistivity varies nonmonotonously with T, and even changes sign, thus violating the conventional expression (Eq. 1). Additionally, we observe a small S-like feature below 100 K and superimposed S-like feature above it, indicating the presence of an anomalous Hall effect (AHE) contribution. While the ordinary Hall coefficient 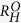 = *dR*_*xy*_/*dH* from the slope of both the two states shows dependent of temperature and field [the slope at different temperature and magnetic field differ], the slope varies with temperature and even changes sign at about 50 K. In both polarization directions, we observed a large AHE signal and the magnetic field dependence of the Hall resistivity clearly deviates from eq.(1). However, it was difficult to determine the normal and anomalous Hall contributions because the AHE did not saturate even at 13 T. Therefore, the normal and anomalous Hall contributions are temperature and magnetic field dependence. Although the separation process between the normal and anomalous Hall contributions is arbitrary, our results suggest that the value and the T-dependence of Hall coefficient are totally different between the two polarization directions. The ordinary coefficient at 1 T and the AHE resistance contribution *R*_AH_ from linear fits to H = 13 T increases sharply with decreasing temperature, and even changes sign at about 50 K as shown in Fig. 3(e,f). Once again, there is a stark contrast between UPS and DPS, for one hand, the absolutely of ordinary Hall coefficient 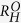
in UPS is larger than in DPS below 50 K but smaller above it. Besides, the absolutely of AHE resistance *R*_*AH*_ in UPS and DPS shows the same behaviors. The S-like feature in the Hall curves in Fig. 3 at intermediate temperatures such as below 100 K show oscillatory behavior implies that the AHE is composed of positive and negative components with different saturation magnetic fields. Therefore, the Hall resistance should consist of three components: the ordinary Hall resistivity, a positive component and a negative component. It is clearly seen that the AHE is tuned by polarization direction and shows a sign change between low and high temperatures. The sign change of AHE as a function of T has been observed in several materials including Pt, contacted with FM, such as yttrium iron garnet(YIG)^4^,^5^,^18^,^19^.This T dependence has been interpreted in terms of a singularity in the band structure of Pt, which can be regarded as a magnetic monopole in momentum space^20^. Hence, one possible reason is that the introduction of magnetic ordering in Pt by the BLFO underlayer will result in the band split for the Pt 5d spin-up and spin-down electrons, which in turn lower the density of states (DOS) at the Pt Fermi level. Reduction in the DOS will result in less s-d scattering and lead to a smaller resistivity for the magnetic ordered Pt layer.

Therefore, comparison between the Pt film resistivity of UPS and DPS also suggests that the electronic structure of the Pt layer has been appreciably modified by the BLFO underlayer. Hence, we argue that the different Hall effect behaviors in our results may be resulted from the different proximity effect between DPS and UPS interface.

Phenomenologically, we believe the interfacial electrons from Pt can penetrate into BLFO and be reflected back into the Pt. Affected by the strong exchange interaction within BLFO, spin splitting and appreciable change in the DOS at the Fermi level will happen in the interfacial Pt layers, and, thus, lead to the observed thickness-dependent Pt transport behavior. The details of the phenomenon are currently under theoretical investigation. Because of the ferroelectric nature of BLFO, when ferroelectric polarization is switched, the Fe and Bi ions will move relative to the oxygen octahedra^21^. Since the Pt layer does not exhibit the same effect, the Fe ion at the interface in BLFO exists in two different states depending on FE polarization, one closer to the Pt layer and one farther away. By changing the distance between interacting ions, we are manipulating interfacial interaction energy to cause two different magnetic states to exist: the low interaction(positive voltage pulse, down polarization, weak magnetic proximity effect, Fe closer to interface) state and the high interaction (negative gate voltage pulse, up polarization, strong magnetic proximity effect, Fe farther from interface) state.

**Figure 4:**
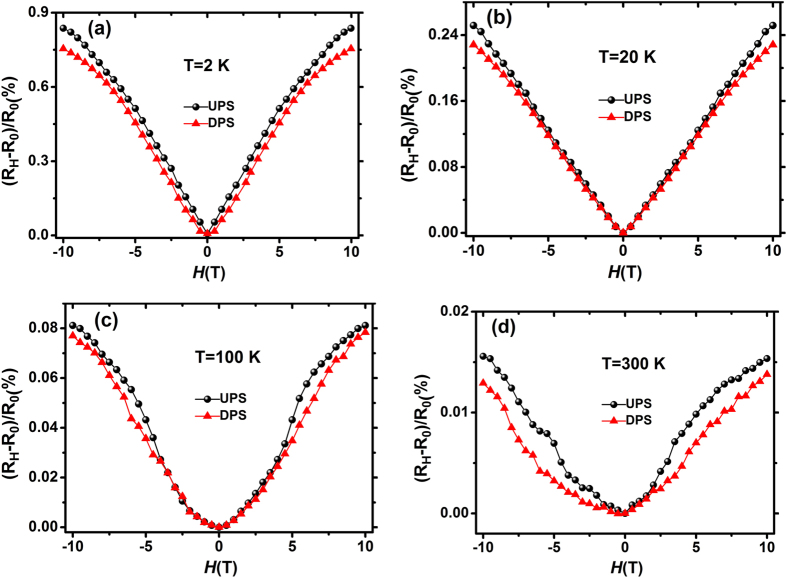
Magnetic field (*H*) dependence of magnetoresistance (*R_H_-R_0_*)/R_0_ in H//z direction forPt/BLFO films with upward polarization state and downward polarization state. (**a**) Magnetic field (*H*) dependence of magnetoresistance (*R_H_-R_0_*)/*R_0_* in H//z direction for Pt/BLFO films with upward polarization state and downward polarization state at 2 K. (**b**) Magnetic field (*H*) dependence of magnetoresistance (*R_H_-R_0_*)/*R_0_* in H//z direction for Pt/BLFO films with upward polarization state and downward polarization state at 20 K. (**c**) Magnetic field (*H*) dependence of magnetoresistance (*R_H_-R_0_*)/*R_0_* in H//z direction for Pt/BLFO films with upward polarization state and downward polarization state at 100 K. (**d**) Magnetic field (*H*) dependence of magnetoresistance (*R_H_-R_0_*)/*R_0_* in H//z direction for Pt/BLFO films with upward polarization state and downward polarization state at 300K.

Figure 4 shows magnetic field (H) dependence of MR for Pt/BLFO at different temperatures with BLFO films in UPS and DPS. Here, the magnitude of MR is defined as (*R*_*H*_-*R*_*0*_)/*R*_*0*_. A positive MR is observed, and its magnitude is ~0.85% and ~0.75% at 13 T and 2 K for UPS and DPS, respectively. This positive MR decreases monotonously with temperature increase, as shown from Fig. 4(a–d).This positive MR effect is usually attributed to weak anti-localization (WAL) which appears in disordered conductors with strong spin-orbit coupling^22^,^23^,^24^,^25^,^26^. The magnitude of change between MR ratio and the Hall resistance are not the same, this may be that the conductivity change is expected to be proportional to the ratio of the polarization induced carrier density to the initial sheet carrier density, and the initial resistance between Rxx and Rxy is different. This different behavior has also been observed in Gate tuning of anomalous Hall effect in ferromagnetic metal SrRuO_3_.We compare the T range of MR enhancement and that of weak anti-localization regime determined from R-T curve (Fig. 2). We found that in the weak anti-localization regime, positive MR at 10 T is reduced almost in proportion to *ln*T, which signals weak anti-localization (WAL) behavior, indicating that the resistance rise in Fig. 2 and WAL in Fig. 4 are related with each other.

**Figure 5:**
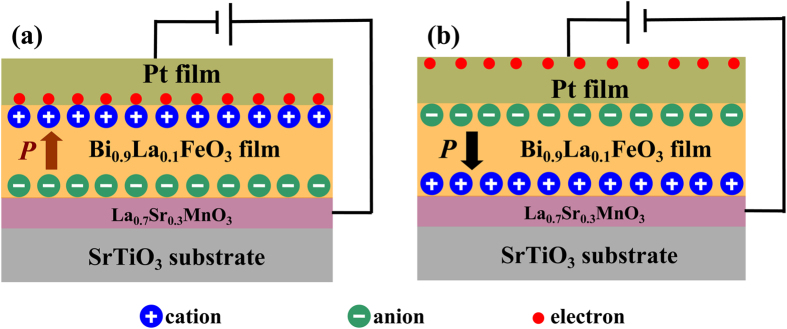
The schematic pictures of the two configurations. (**a**) The schematic pictures of the two configurations for upward polarization state. (**b**) The schematic pictures of the two configurations for downward polarization state. Different polarization direction will differ local field, which in turn cause different effect on electrons in Pt, i.e., upward polarization direction of BLFO films producing downward electric field and thus can attract electrons while the electrons was repelled away from BLFO interface with downward polarization direction. Both structures induce an AHE, as shown in Fig. 3 (a)–3(d), respectively.

The mechanism of the induced anomalous Hall effect remains the subject of ongoing investigation. A possible scenario is that BLFO ferroelectric films may cause a local electric fields at the Pt/BLFO interface,which might induce local magnetic moments in Pt with the help of its large spin-orbit interaction. The local electric field caused by BLFO films can be estimated to be ~400 V/nm according to the formula ***E*** = *σ*/2ε_0_ for infinite plate. Where *σ and* ε_0_ are the charge density of BFLO films surface (~80 μC/cm^2^, as shown in Fig. 3(c)) and the permittivity of vacuum. Therefore, different polarization direction will differ local field,which in turn cause different effect on electrons in Pt, i.e., upward polarization direction of BLFO films producing downward electric field and thus can attract electrons while the electrons was repelled away from BLFO interface with downward polarization direction, the schematic pictures of a comparison of both the upward and downward polarization directions are showed in Fig. 5. This electric field caused AHE is in accord with the recent report by Shimizu *et al*.^27^, where the nonlinear Hall resistance was attributed to the strong electric field produced at the electric double layer interface between ions and electron conductors. However, further investigation of the possible source of the induced magnetism in the polarization tuned Pt thin films is still needed.

## Conclusions

In summary, the electrical transport characteristics of thin Pt films on a antiferromagnetic semiconductor BLFO films was studied. We have discovered a mechanism for the direct control of Hall effect with electric field. This effect is reversible and comes concurrently with the modulation of magnetic proximity effect and the modulation of resistivity. We have demonstrated polarization direction tuning of the AHE in nonmagnetic Pt thin films, indicating a common underlying mechanism. Thus, the polarization direction tuning of the AHE in paramagnetic metal Pt has further revealed a novel aspect of electronic and magnetic properties of Pt thin films. This type of Hall effect control is a crucial first step for fully controlling the magnetization of a thin film using an electric field. Using this method of controlling magnetization and Hall effect would offer a low-current or low-power alternative to the typical current induced magnetization control mechanisms. From both a device applications and physics standpoint, these results represent an exciting advance for the next generation of Hall effect systems and devices.

## Additional Information

**How to cite this article**: Gao, R. *et al*. Electric Control of the Hall effect in Pt/Bi_0.9_La_0.1_FeO_3_ bilayers. *Sci. Rep*. **6**, 20330; doi: 10.1038/srep20330 (2016).
